# Identifying the Related Genes of Muscle Growth and Exploring the Functions by Compensatory Growth in Mandarin Fish (*Siniperca chuatsi*)

**DOI:** 10.3389/fphys.2020.553563

**Published:** 2020-09-25

**Authors:** Xuange Liu, Shuang Zeng, Shuang Liu, Gongpei Wang, Han Lai, Xiaopin Zhao, Sheng Bi, Dingli Guo, Xiaoli Chen, Huadong Yi, Yuqin Su, Yong Zhang, Guifeng Li

**Affiliations:** ^1^Guangdong Province Key Laboratory for Aquatic Economic Animals, School of Life Sciences, Sun Yat-sen University, Guangzhou, China; ^2^Guangdong Provincial Engineering Technology Research Center for Healthy Breeding of Important Economic Fish, Guangzhou, China; ^3^Southern Marine Science and Engineering Guangdong Laboratory, Zhuhai, China

**Keywords:** mandarin fish, RNA-seq, compensatory growth, muscle growth-related genes, gene expression

## Abstract

How organisms display many different biochemical, physiological processes through genes expression and regulatory mechanisms affecting muscle growth is a central issue in growth and development. In *Siniperca chuatsi*, the growth-related genes and underlying relevant mechanisms are poorly understood, especially for difference of body sizes and compensatory growth performance. Muscle from 3-month old individuals of different sizes was used for transcriptome analysis. Results showed that 8,942 different expression genes (DEGs) were identified after calculating the RPKM. The DEGs involved in GH-IGF pathways, protein synthesis, ribosome synthesis and energy metabolisms, which were expressed significantly higher in small individuals (S) than large fish (L). In repletion feeding and compensatory growth experiments, eight more significant DEGs were used for further research (GHR2, IGFR1, 4ebp, Mhc, Mlc, Myf6, MyoD, troponin). When food was plentiful, eight genes participated in and promoted growth and muscle synthesis, respectively. Starvation can be shown to inhibit the expression of Mhc, Mlc and troponin, and high expression of GHR2, IGFR1, and 4ebp inhibited growth. Fasting promoted the metabolic actions of GHR2, IGFR1, and 4ebp rather than the growth-promoting actions. MyoD can sense and regulate the hunger, which also worked with Mhc and Mlc to accelerate the compensatory growth of *S. chuatsi*. This study is helpful to understand the regulation mechanisms of muscle growth-related genes. The elected genes will contribute to the selective breeding in future as candidate genes.

## Introduction

Muscle growth is regulated by the gene expression nets of muscle cell proliferation and protein metabolism (Riley et al., 2002). In vertebrates, several growth-related genes have been identified, including MRFs, IGF, IGFR, GH, GHR (De-Santis and Jerry, 2007; Devlin et al., 2009). Interestingly, for mammals, muscle growth occurs mainly by hypertrophy, with little proliferation. Unlike mammals, fish muscle growth is achieved by hyperplasia and hypertrophy (Rowlerson and Veggetti, 2001). Furthermore, in large and fast-growing fish, both hyperplasia and hypertrophy contribute to muscle growth and continue into a large body size, whereas small and slow-growing fish largely rely on hypertrophy and the rate of muscle fiber recruitment is rather low (Veggetti et al., 1993; Steinbacher et al., 2010; Weatherley et al., 2010). Thus, the same genes may have different expression and regulatory mechanisms in different fish. Studies on genes expression of different sizes and compensatory growth prove fruitful for comprehending growth regulatory mechanisms of fish (Chauvigné et al., 2003; Nebo et al., 2013; Picha et al., 2014; Tian et al., 2016).

Body size is an obvious and vital characteristic of fish, which is controlled by cell number and cell size (Brogiolo et al., 2001), and this process is tightly modulated by growth-related genes and nutrition. An adequate re-feeding following a period of starvation or unfavorable environmental conditions can result in accelerated growth, which is called compensatory growth and has been widely studied in vertebrates (Ali et al., 2003; Chauvigné et al., 2003; Montserrat et al., 2007; Nebo et al., 2013). Especially, it has been reported that fish has the capacity of compensatory growth (Ali et al., 2003). In the meantime, transcriptome analysis can identify growth-related genes and expand knowledge in body size and compensatory growth, promoting the enhanced rate of food utilization and cut costs in aquaculture industry.

Transcriptome and RNA-sequencing (RNA-seq) have been applied to a substantial amount of fish biology studies, including zebrafish (Collins et al., 2012), channel catfish (Liu et al., 2012), European sea bass (Sarropoulou et al., 2012), and rainbow trout (Palstra et al., 2013). Many biological processes, including development, immune, stress response, and adaptive evolution can be mapped, annotated and understood by RNA-seq.

*Siniperca chuatsi* is an important commercial fresh water fish in China. At present, the research on *S. chuatsi* mainly focuses on aquaculture, disease and immunity, nevertheless the growth-related genes and mechanisms are not well-understood (Wu et al., 2015; Huang et al., 2017; Dou et al., 2018). To this end, different size muscle of *S. chuatsi* was subjected to transcriptome analysis by RNA-seq and the mechanisms of growth-related genes by compensatory growth in this study. The results would be helpful to find significantly molecule markers for selective breeding.

## Materials and Methods

### Ethics Statement

Mandarin fish experiments were approved by the Institutional Animal Care and Ethics Committee of Sun Yat-sen University and performed in accordance with the guidelines for experimental animals established by this committee.

### Experimental Animals

In this study, all of *S. chuatsi* came from Guangdong Foshan Bairong Aquatic Breeding, Co., Ltd. Three full-sib families A, B, and C were constructed by artificial insemination (Figure 8). Each family contained 80 *S. chuatsi* in a separate tank. The experimental fish were fed under uniform conditions; they were provided adequate live fish as bait twice daily at 0900 and 1800 h. The water temperature was maintained at 25–26°C. After feeding 3 months in family A, 5S and 5L was performed for RNA-seq. For B and C families, 6L and 6S fish were selected from each family at 3- and 5-month old for real-time PCR (One of the six fish was chosen as a standby) (Figure 8).

### Tissue Sampling

All of the above experimental fish were anesthetized with tricaine methanesulfonate (MS-222, 100 mg/L) and sacrificed via decapitation for subsequent sampling, and white muscle was removed immediately. The tissues were frozen in liquid nitrogen quickly, and stored at −80°C until use. Furthermore, each experiment was performed with three independent biological replicates.

### RNA Extraction and Library Construction

For RNA-seq, total RNA was extracted from white muscle in Family A with E.Z.N.A. total RNA kit II and detected with the concentration and quality. To acquire the entire transcriptome information, and to find out the growth-related DEGs, the muscle samples of family A were mixed with equal amount and then were divided into two RNA pools. PolyA mRNA was isolated by Beads with Oligo (dT) after total RNA was collected and interrupted to short fragments. Random hexamer-primer was used to synthesize the first-strand cDNA using the Qiaquick PCR Purification Kit (Qiagen). The second-strand cDNA was synthesized using buffer, dNTPs, RNaseH and DNA polymerase I, respectively (Invitrogen). Subsequently, short fragments were purified, enriched for end reparation and adding polyA, connected with sequencing adapters. After that, the suitable fragments were selected using agarose gel electrophoresis for the PCR amplification as templates. At last, the two cDNA library could be sequenced in BGI-Shenzhen using Illumina HiSeq^TM^ 2000.

### Illumina Reads Processing and Assembly

Clean reads were screened from raw reads gained from sequencing machines by removing adaptors, unknown nucleotides larger than 5% and low quality reads (which the percentage of low Q-value ≤ 10 base was more than 20%) which would negatively affect following bioinformatics analysis. Firstly, program-Trinity combines reads with certain length of overlap to form longer fragments without N, which are called contigs. Then, these contigs were taken into further process of sequence cluster with sequence clustering software to form longer sequences without N, which are defined as unigenes.

### Functional Annotation and Classification

The unigenes sequences were firstly aligned with a series of public databases, such as the non-redundant protein database, the Cluster of Orthologous Groups (COG) of protein database, the Kyoto Encyclopedia of Genes and GenBank non-redundant (NR) database and Swiss-Prot database, using BLASTx (E-valueb10-5) and BLAST (E-valueb10-10), respectively. According to the Nr annotation information, the obtained unigene was enriched with Gene Ontology (GO) by Blast2GO program. And then unigenes were classified into different GO functional cluster using WEGO software. GO has three ontologies: molecular function, cellular component and biological process. The basic unit of GO is GO-term, and every GO-term belongs to a type of ontology.

### Differential Genes Expression Analysis

The genes expression was calculated by the numbers of reads that mapped to the reference sequence and every gene. All genes expression levels were calculated by using the formula RPKM method and the RPKM of genes can be used for comparing between different samples. Different expression genes were carried out GO function analysis to understand its function. False discovery rate (FDR) was used to determine the threshold of *P*-value which corresponds to differential gene expression test in multiple tests. FDR ≤ 0.001 and the absolute value of log_2_^Ratio^ ≥ 1 is used as the threshold to judge the significance of gene expression difference.

### The Synthesis of cDNA and Real-Time PCR Detection

cDNA was synthesized from muscle in B and C families with PrimeScript^®^RT reagent Kit with gDNA Eraser (Perfect Real Time). The specific primers for the RT-PCR were designed based on selected growth-related genes using the Primer Premier5.0. Corresponding primers were designed to vertify the results (Table 5). 6 DEGs were selected as the verification genes to confirm the reliability of data obtained by RNA-seq. And 18SrRNA was used as a reference gene which was no evident difference in different samples of *S. chuatsi*. The primers were listed in Table 5. Quantitative Fluorescence PCRassay was performed with LightCycler^®^ 480 II Real-Time PCR System (Roche), and the kits were SYBR^®^ Premix Ex TaqTM II (Tli RNaseH Plus) (TaKaRa, Japan), with 384-well plates. In each reaction system, there were 10 ul including the first-strand cDNA, 250 nM primers, and 5 μl SYBR Green PCR Master Mix (TaKaRa, Japan). Each reaction was repeated three times. And the relative expression levels were calculated by the 2^–ΔCt^ method.

### Fasting and Re-feeding Experiments

Since transcriptome results represented gene expression at a certain time, change of gene expression over time cannot be known. So we researched the change of gene expression and its mechanism in compensatory growth experiments. A total of 80 experimental fish from one family (300 ± 30 g, body weight) were randomly divided into control and experimental groups that each included four repeated subgroups, and they were provided live fish as bait. Each subgroup contained 20 in a separate tank. The water temperature was maintained at 25–26°C, and the compensatory growth experiment lasted for 6 weeks. During an initial 3 weeks of fasting, food was withheld from the experimental group, while that of the control group was maintained at a normal level. After this, both the experimental group and the control group were fed continuously during the following 3 weeks. Seven sampling time points (0–6) were set throughout the experiment. At each sampling time point, six *S. chuatsi* in a pond were randomly selected from each group and sampled following anesthesia with MS-222 (100 mg/L). Sampling time points 0, 1, 2, and 3 represented *S. chuatsi* that had been fasted for 0, 1, 2, and 3 weeks, respectively. Sampling time points 4, 5, and 6 represented the fish that were re-fed continuously for 1, 2, and 3 weeks after starvation, respectively. The fish were fed twice a day and weighed every week, and weights were used to calculated The Specific growth rate (SGR). SGR means ((In W 2 -W 1)/(T 2 -T 1) × 100), where W 2 is the weight at the end of the growth interval and W 1 is the weight at the beginning of the growth interval, while T 2 -T 1 represents the duration (days) of the growing interval. The white muscle samples collected from *S. chuatsi* was rapidly frozen in liquid nitrogen and stored at −80°C and samples were used for real-time PCR analysis.

### Statistical Analyses

SPSS version 18.0 was used for the analyses in the study, Tukey’s test was applied to compare the significant differences of the expression of the growth-related genes. The level of significance for all statistical tests was set at 5% (*P* < 0.05).

## Results

### Sequencing and Annotation

**FIGURE 1:**
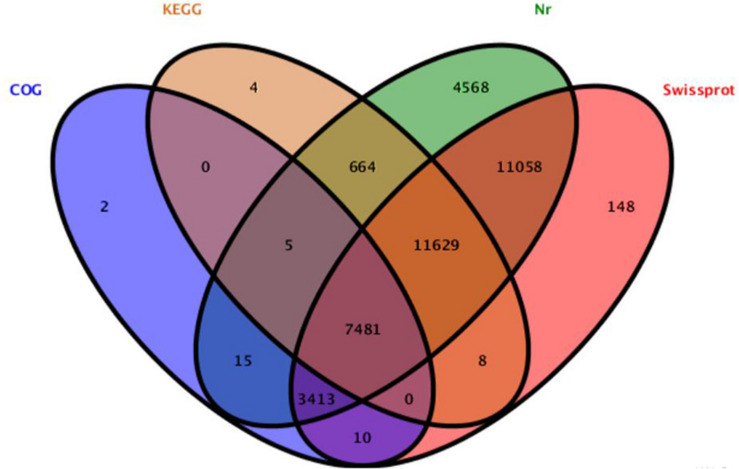
Detection of homologous genes in public databases. The numbers of annotated all-unigenes were indicated in the ellipses respectively.

The different weight of *S. chuatsi* was shown in Table 1. The brief information of Illumina deep sequencing was shown in Table 2. A total of 91,264,026 high quality clean reads (48963598 belongs L, 46617484 belongs S) were obtained (Table 2). After screening, 39005 unigenes were annotated into four databases, including Nr (38833, 52.9%), Swissprot (337479, 46.0%), COG (10926, 14.8%), and KEGG (19791, 26.9%) (Figure 1). So, we annotated the results by Nr database which defined the maximum number of unigenes.

**TABLE 1:** Growth traits of small and large *Siniperca chuatsi.*

Individual	Weight (g)	Length (mm)	Height (mm)
L-1	62.2	138	46
L-2	80.1	147	52
L-3	55.2	132	43
L-4	54.3	131	45
L-5	59.3	135	44
S-1	12.6	79	24
S-2	7.8	71	23
S-3	16	85	26
S-4	12.9	82	24
S-5	12.8	79	26

*L means Large individual, S means Small individual.*

**TABLE 2:** The summary information of deep sequencing and assembly for small and large *Siniperca chuatsi.*

Category	Number
Number of reads	95,581,082
Clean reads	91,264,026 (95.48%)
Total nucleotides (nt)	9,126,402,600
Q20 ratio (%)	98.57
N ratio (%)	0.00
GC ratio (%)	51.31
Number of contigs	80,314
Average length of unigenes	621
Number of unigenes	73,353
Average length of unigenes (bp)	703

### Analysis of RNA-seq Data and Confirmation

**FIGURE 2:**
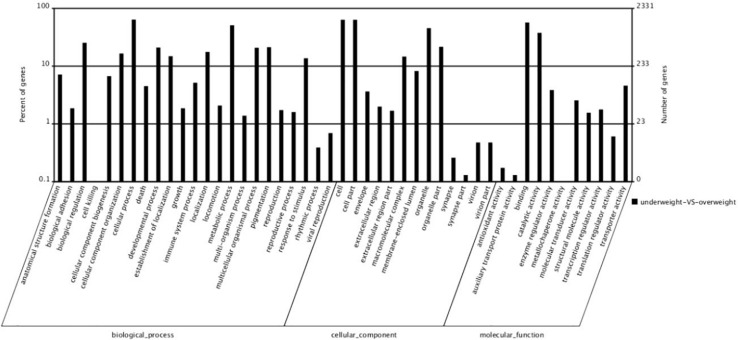
The number of different expression genes that the genes of large fish compared to the genes of small fish.

**FIGURE 3:**
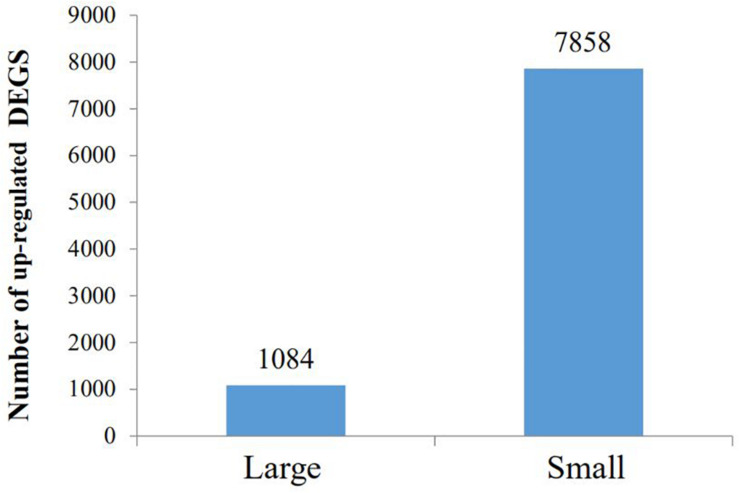
The GO analysis of different expression genes. The 5150 differentially expressed genes were divided into three groups, respectively, biological process (1530), cellular component (1737), and molecular function (1883).

By GO terms enrichment analysis, the biological process, cellular component and molecular function have 1530, 1737, and 1883 genes respectively (Figure 2), which illustrated the dominant groups was cellular process in biological process, cell and cell part in cellular component and binding in molecular function (Figure 2). 8,942 DEGs were identified after calculating the RPKM. Among them, 1084 unigenes were up-regulated in L, while 7858 unigenes were up-regulated in S (Figure 3). The results showed that more genes were up-regulated in S compared to L (Figure 3), including GH-IGF pathways receptors and downstream genes (GHR2, IGFR1, 4ebp, P70S6K, transcriptional elongation factor, etc.), muscle synthesis and contraction genes (Mhc, Mlc, troponin, etc.) and energy metabolism genes (Glucose-6 phosphate dehydrogenase, etc.) (Table 3). Thus, eight genes were selected for further research by real-time PCR, involving GHR2, IGFR1, 4ebp, MHC, Mlc, MyoD, Myf6, and troponin.

**TABLE 3:** The different expression genes between small and large *Siniperca chuatsi.*

Gene ID	Annotation	Small fish RPKM	Large fish RPKM	log2 Ratio	*P*-value	FDR
**IGF system**						
Unigene0026866	Type 1 insulin-like growth factor receptor	1.517	0.001	−10.567	6.09E-07	5.42E-06
Unigene0012575	Insulin-like growth factor 2 receptor	3.936	0.891	−2.143	1.54E-16	3.34E-15
Unigene0054506	Insulin receptor substrate 2-like	20.124	5.191	−1.955	3.20E-276	5.24E-274
Unigene0029023	Insulin-like growth factor binding protein-1	7.535	3.251	−1.213	2.69E-30	9.85E-29
Unigene0010948	Ribosomal protein S6 kinase beta-1	0.959	0.048	−4.309	1.39E-05	9.96E-05
Unigene0047933	Ribosomal protein S6 kinase alpha-1 isoform X1	4.687	1.575	−1.573	2.11E-07	2.01E-06
Unigene0038236	Ribosomal protein S6 kinase alpha-6,	0.521	0.125	−2.061	1.04E-07	1.03E-06
Unigene0060291	Eukaryotic translation initiation factor 4E-binding protein	45.133	5.536	−3.027	3.95E-216	5.91E-214
Unigene0023276	Eukaryotic translation initiation factor 2-alpha kinase 1	0.797	0.001	−9.638	4.43E-05	2.84E-04
Unigene0056547	Elongation factor Tu GTP-binding domain-containing protein 1 isoform 2	1.813	0.404	−2.165	1.61E-06	1.35E-05
Unigene0059231	Elongation factor Tu GTP-binding domain-containing protein 1 isoform X1	1.337	0.427	−1.646	4.45E-05	2.85E-04
Unigene0052980	GTP-binding protein Rheb	17.230	8.132	−1.083	2.72E-41	1.31E-39
Unigene0037630	Ras-related protein Rap-1A	0.712	0.246	−1.535	4.21E-05	2.74E-04
Unigene0059752	MAPK kinase-interacting serine/threonine-protein kinase 1 isoform X1	6.727	2.459	−1.452	8.03E-29	2.83E-27
Unigene0036862	TGF-beta-activated kinase 1 and MAP3K7-binding protein 1	5.344	1.770	−1.594	2.14E-27	7.19E-26
Unigene0071651	Mitogen-activated protein kinase kinase kinase 5	3.057	1.302	−1.231	1.48E-13	2.63E-12
Unigene0074102	Growth hormone receptor isoform 1	23.867	4.137	−2.528	5.05E-164	6.66E-162
**Protein synthesis (myosin)**						
Unigene0068992	Myosin light chain 1	3.163	0.253	−3.646	3.44E-06	2.74E-05
Unigene0068993	Myosin light chain 1	6.423	0.058	−6.789	6.95E-32	2.66E-30
Unigene0008763	Slow myosin heavy chain 1	1.017	0.334	−1.609	1.61E-07	1.55E-06
Unigene0032403	Tropomyosin alpha-1 chain	2.572	0.183	−3.816	4.96E-07	4.48E-06
Unigene0021259	Myosin heavy chain, fast skeletal muscle-like	4.050	0.578	−2.808	5.39E-09	6.18E-08
Unigene0002916	Actin	5.893	0.741	−2.991	5.70E-12	8.74E-11
Unigene0058346	Activin receptor type-1B	2.769	0.992	−1.481	6.59E-08	6.70E-07
Unigene0023112	Myogenic factor 6	8.409	3.309	1.3464	1.94E-21	5.25E-20
**Ribosome systhesis**						
Unigene0021505	WD repeat-containing protein 3	3.780	0.617	2.616	1.37E-15	2.81E-14
Unigene0028009	Ribonuclease P protein subunit p40	5.950	1.123	−2.405	8.85E-64	6.18E-62
Unigene0028767	U3 small nucleolar RNA-associated protein 18 homolog	4.367	0.976	−2.162	5.92E-37	2.57E-35
Unigene0038257	Ribosomal RNA small subunit methyltransferase NEP1	5.854	1.982	−1.562	5.71E-17	1.26E-15
Unigene0025207	Ribosome maturation protein SBDS	13.361	4.897	−1.448	1.05E-59	7.00E-58
Unigene0036337	U3 small nucleolar ribonucleoprotein protein IMP3	9.784	4.186	−1.225	2.97E-26	9.59E-25
Unigene0033017	SET and MYND domain-containing protein 1 isoform 2	0.707	0.208	−1.762	4.03E-06	3.17E-05
Unigene0073572	SET and MYND domain-containing protein 5	6.901	3.190	−1.113	8.29E-21	2.19E-19
**Energy metabolism**						
Unigene0047705	Hexokinase	3.809	1.657	1.201	6.71E-15	1.32E-13
Unigene0047702	Hexokinase-2	11.175	1.254	−3.156	5.87E-39	2.67E-37
Unigene0012253	Pyruvate dehydrogenase kinase isozyme 1, mitochondrial isoform 4	2.384	1.031	−1.209	1.37E-10	1.83E-09
Unigene0038645	Acyl-CoA synthetase long-chain family member 4	1.721	0.458	−1.909	5.43E-05	3.43E-04
Unigene0066639	Acyl-CoA dehydrogenase family member 11	1.034	0.384	−1.428	1.22E-04	7.11E-04
Unigene0048024	Short-chain specific acyl-CoA dehydrogenase, mitochondrial precursor	7.953	3.562	−1.159	2.99E-56	1.89E-54
Unigene0053754	Heat shock protein 70	3.978	0.168	−4.564	6.41E-18	1.48E-16
Unigene0022967	Heat shock protein hsp90 beta	8.095	1.880	−2.106	6.28E-95	5.87E-93
Unigene0029204	Heat shock protein HSP 90-alpha	15.907	7.477	−1.089	6.52E-13	1.09E-11
**Protein degradation**						
Unigene0064302	Cathepsin O	3.062	1.468	−1.061	1.07E-07	1.06E-06
Unigene0054503	Cathepsin F	25.301	6.568	−1.946	4.47E-161	5.86E-159
Unigene0069862	Calpain-1 catalytic subunit	0.424	0.119	−1.827	2.86E-06	2.30E-05
Unigene0065029	Calpain-3	3.497	0.319	−3.453	7.02E-14	1.29E-12
Unigene0029262	Calpain 5	3.229	1.140	−1.502	6.28E-07	5.58E-06
Unigene0064726	E3 ubiquitin-protein ligase CBL	1.302	0.288	−2.177	4.70E-06	3.66E-05
Unigene0047859	Ubiquitin carboxyl-terminal hydrolase CYLD isoform X1	1.511	0.426	−1.827	1.42E-04	8.18E-04
nigene0048496	E3 ubiquitin-protein ligase SIAH1	1.643	0.545	−1.594	7.69E-08	7.72E-07
Unigene0037497	Ubiquitin carboxyl-terminal hydrolase 8	3.049	1.497	−1.026	3.54E-16	7.52E-15
Unigene0006529	Ubiquitin-conjugating enzyme E2R 2	13.000	2.437	−2.415	3.04E-51	1.77E-49

**FIGURE 4:**
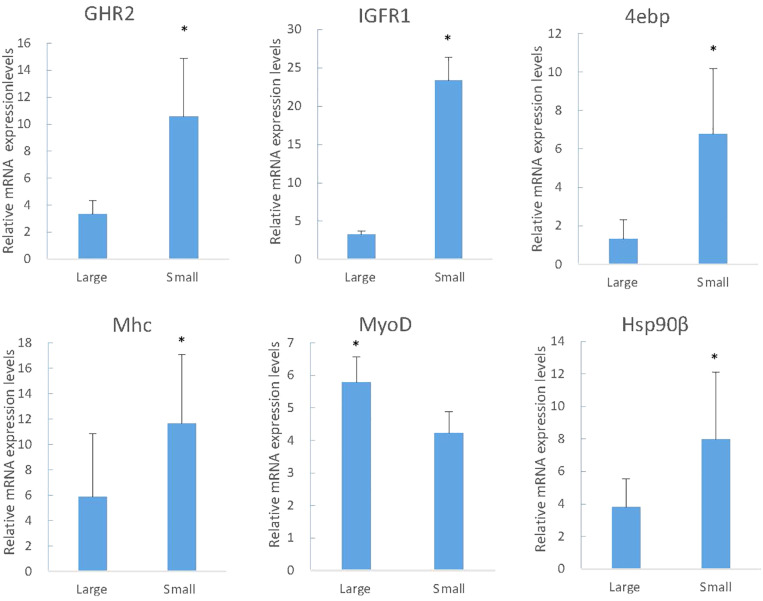
The RT-PCR analysis of gene expression in large and small fish. The y-axis indicates relative expression level between the samples of large and small fish using the Relative Quantitation. IGFR1, insulin-like growth factor receptor -1; GHR1, growth factor receptor-1; 4EBP, eukaryotic translation initiation factor 4E-binding protein; Mhc, fast skeletal muscle myosin heavy chain; Hsp90β, heat shock protein hsp90 beta (Hsp 90β); MyoD, myoblast determination protein. Significant differences at the *P* < 0.05 level are indicated by * above the columns.

KEGG analysis obtained 25 pathways, among which the most differentially expressed genes were protein processing in endoplasmic reticulum (119, 3.77%), ubiquitin mediated proteolysis (101, 3.2%) and RNA transport (91, 2.88%), respectively (Table 4). The results were confirmed by RT-PCR, proving that the genes expression difference and transcriptome results had the same trend (Figure 4).

**TABLE 4:** The pathway analysis of different expression genes between small and large in *Siniperca chuatsi.*

Pathway ID	Pathway	DEGs with pathway annotation	All genes with pathway annotation	*P*-value
ko03050	Proteasome	42(1.33%)	73(0.37%)	5.69E-16
ko03008	Ribosome biogenesis in eukaryotes	62(1.96%)	168(0.85%)	3.04E-11
ko04141	Protein processing in endoplasmic reticulum	119(3.77%)	435(2.2%)	7.21E-10
ko04120	Ubiquitin mediated proteolysis	101(3.2%)	357(1.8%)	2.01E-09
ko00240	Pyrimidine metabolism	58(1.84%)	189(0.95%)	2.84E-07
ko04142	Lysosome	78(2.47%)	282(1.42%)	3.67E-07
ko04140	Regulation of autophagy	20(0.63%)	43(0.22%)	2.44E-06
ko03030	DNA replication	24(0.76%)	60(0.3%)	6.70E-06
ko03430	Mismatch repair	20(0.63%)	46(0.23%)	8.78E-06
ko03420	Nucleotide excision repair	28(0.89%)	78(0.39%)	1.38E-05
ko03013	RNA transport	91(2.88%)	383(1.94%)	4.08E-05
ko04974	Protein digestion and absorption	44(1.39%)	177(0.89%)	0.001422099
ko03410	Base excision repair	20(0.63%)	66(0.33%)	0.002557437
ko00860	Porphyrin and chlorophyll metabolism	17(0.54%)	54(0.27%)	0.003361358
ko03020	RNA polymerase	18(0.57%)	60(0.3%)	0.004613781
ko04130	SNARE interactions in vesicular transport	14(0.44%)	45(0.23%)	0.008341413
ko00230	Purine metabolism	71(2.25%)	341(1.72%)	0.009785118
ko05414	Dilated cardiomyopathy	80(2.53%)	398(2.01%)	0.01530128
ko03040	Spliceosome	71(2.25%)	353(1.78%)	0.02097551
ko00471	D-Glutamine and D-glutamate metabolism	6(0.19%)	15(0.08%)	0.0223566
ko00908	Zeatin biosynthesis	2(0.06%)	2(0.01%)	0.02543888
ko05410	Hypertrophic cardiomyopathy (HCM)	77(2.44%)	393(1.99%)	0.02970835
ko04712	Circadian rhythm – plant	4(0.13%)	9(0.05%)	0.04156557
ko00900	Terpenoid backbone biosynthesis	7(0.22%)	22(0.11%)	0.04928443
ko04920	Adipocytokine signaling pathway	36(1.14%)	172(0.87%)	0.04951438

**TABLE 5:** Primer sequences for Hsp90β, GHR2, IGFR1, MyoD, Mhc, Mlc, Myf6, Troponin, 4ebp, and 18S RNA.

Gene	Primers	Sequences (5′–3′)
Hsp90β	RT-HSP90β-F	CGACTTAGAAACGACTACCACACG
	RT-HSP90β-R	CAGCCTGGAATGCAAAGGTCT
GHR2	RT-GHR2-F	CGCTGCTGAATGTGAGTTTGAC
	RT-GHR2-R	CCCGAACCTCGTGATTGATG
IGFR1	RT-IGFR1-F	GCTACGTGAAGATCCGCCATT
	RT-IGFR1-R	GCTGCAAGTTCTGGTTGTCCA
MyoD	RT-MyoD-F	TTCTCAGAGGCTCCAAACGG
	RT-MyoD-R	GCTCCACGATGCTGGACAGA
Mhc	RT-Mhc-F	TTGTCCGTTGCCTGATTCCTA
	RT-Mhc-R	CTTCCAGCACACCGTTACACC
Mlc	RT-Mlc-F	AACCCCTCCAATGACGACA
	RT-Mlc-R	AATCTCAGGCTCAGTCATCTTCTC
Myf6	RT-Myf6-F	AGACCAACCCTTATCTTTTCAATG
	RT-Myf6-R	CGGTCTCGGACGGAACATTAT
Troponin	RT-Troponin-F	GGGCTCCAAACACACAGTCAAC
	RT-Troponin-R	GCCTTGTCCTCAATGTTCTTACG
4ebp	RT-4ebp-F	TCACCATCCACGATTCTGCTC
	RT-4ebp-R	ACCTCCTGGCGTAGTGCTGA
18S RNA	RT-18S-F	CTGAGAAACGGCTACCACATCC
	RT-18S-R	GCACCAGACTTGCCCTCCA

### The Expression Growth-Related Genes in Different Periods

**FIGURE 5:**
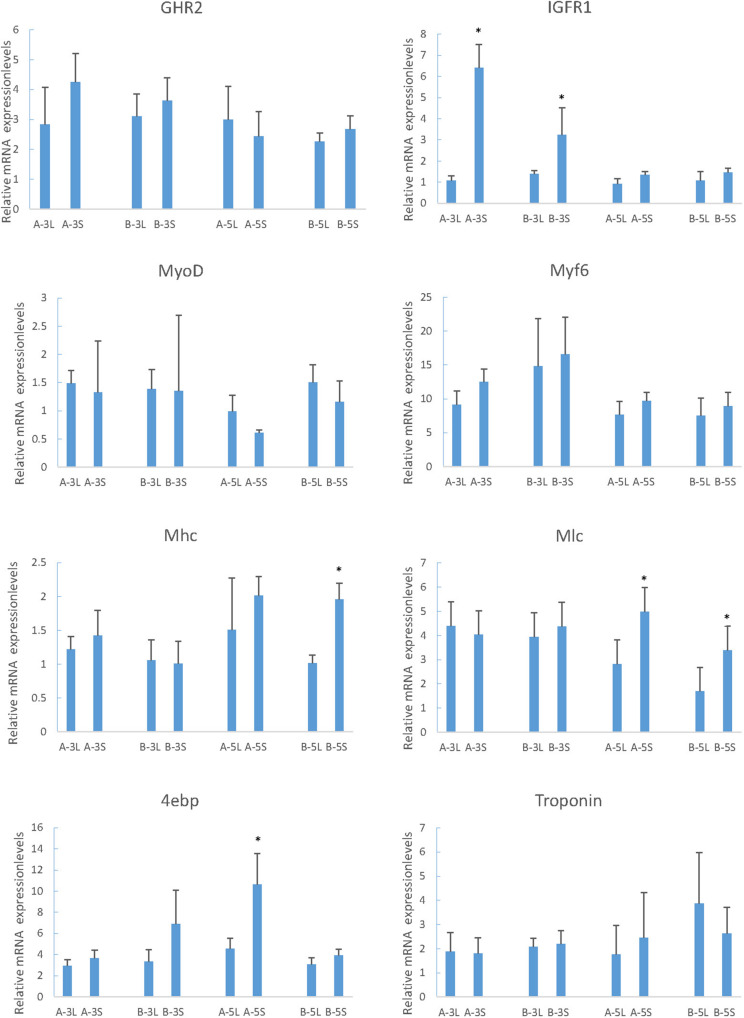
Relative expression of growth-related genes in mandarin fish with different growth traits. 18S was used as a housekeeping gene. All data are represented as the mean ± SEM (*n* = 6). * Indicates a significant (*p* < 0.05) difference between overweight and underweight. A-3L, large fish of 3-month old from A family; A-3S, small fish of 3 months from A family; B-3L, large fish of 3-month old from B family; B-3S, small fish of 3-month old from B family; A-5L, large fish of 5- month old from A family; A-5S, small fish of 5-month old from A family; B-5L, Large fish of 5-month old from B family; B-5S, small fish of 5-month old from B family.

The muscle growth-related genes screened in RNA-Seq were studied in different family and period to identify the expression and regulatory mechanisms. In two periods of two families, the expression of MyoD in L was just above S, but Myf6 was just the opposite. For the expression of IGFR1 and 4ebp in S fish was higher. Along with the increased growth time (5-month old), the expression of Mhc and Mlc in S was significantly higher than that in L (Figure 5, Mhc and Mlc).

### Changes in Body Weight During Compensatory Growth

**FIGURE 6:**
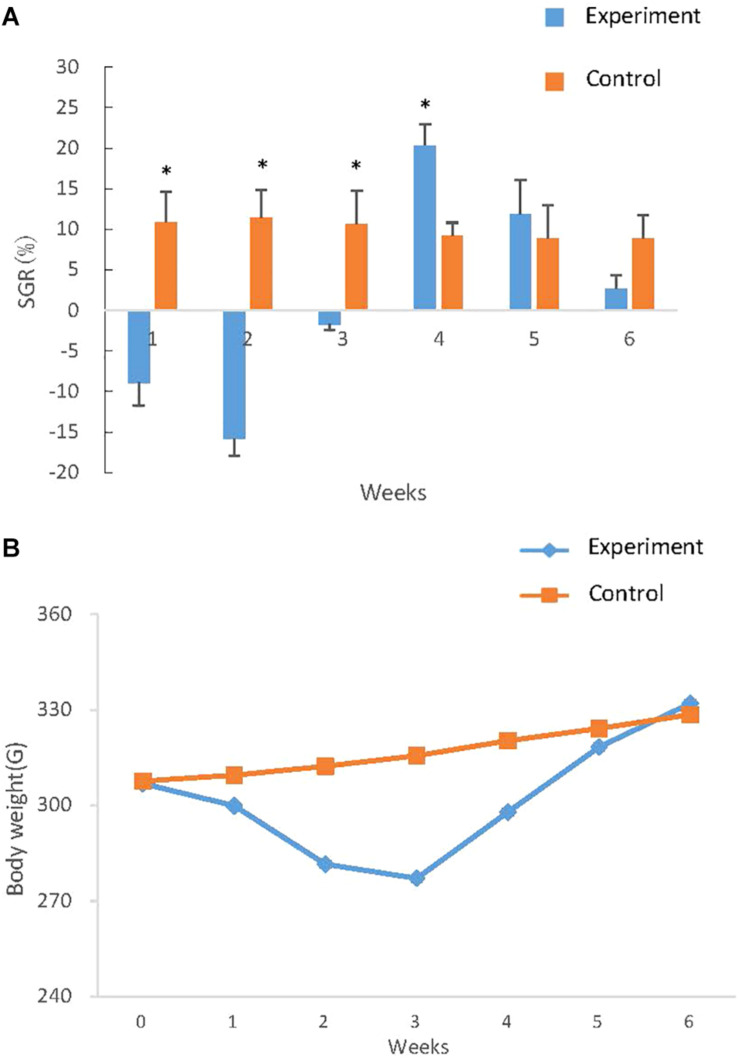
Growth curve and SGR of *S. chuatsi* during compensatory growth. **(A)** Growth curve of *S. chuatsi* during compensatory growth. Fish in experimental and control groups were weighted at six time points and the weights were subjected to curve drawn. In each time point, 6 *S. chuatsi* were random selected and weighted. Data are given as mean ± standard deviation (SD). **(B)** SGR of *S. chuatsi* during compensatory growth. SGRs were calculated for control and experimental group during the six time intervals: the first 3 weeks (1, 2, 3 weeks) means 1, 2, 3 weeks of fasting respectively, the last 3 weeks (4, 5, 6 weeks) means 1, 2, 3 weeks of re-feeding. Asterisks represent significant differences between groups at each time intervals (*P* < 0.05) that calculated by *t-* test.

The SGR in different time intervals was calculated (Figure 6A) and the weight of fish in two groups was reflected at seven time points and curves were drawn (Figure 6B). In the first week, the weight of the experimental group decreased markedly following the induction of starvation, as indicated by a negative SGR (−0.98) which reached the lowest (−2.62) in the second week. In the third week, the body weight loss slowed down (SGR −0.64). During the following week of re-feeding, the weight of the experimental group increased rapidly and resulted in a positive of SGR (2.99), which was significantly higher than (*P* < 0.01) that in the control group. The elevated SGR that characterizes compensatory growth subsequently declined back to low level during the 2 weeks of realimentation, but the SGR of experimental groups was higher than (*P* > 0.05) that in the control group (Figure 6A). The bodyweight difference of experiment and controls ultimately was not significant difference, indicating that the experimental groups achieved complete compensatory growth after re-feeding.

### Effect of Re-feeding to Expression of Related-Growth Genes

**FIGURE 7:**
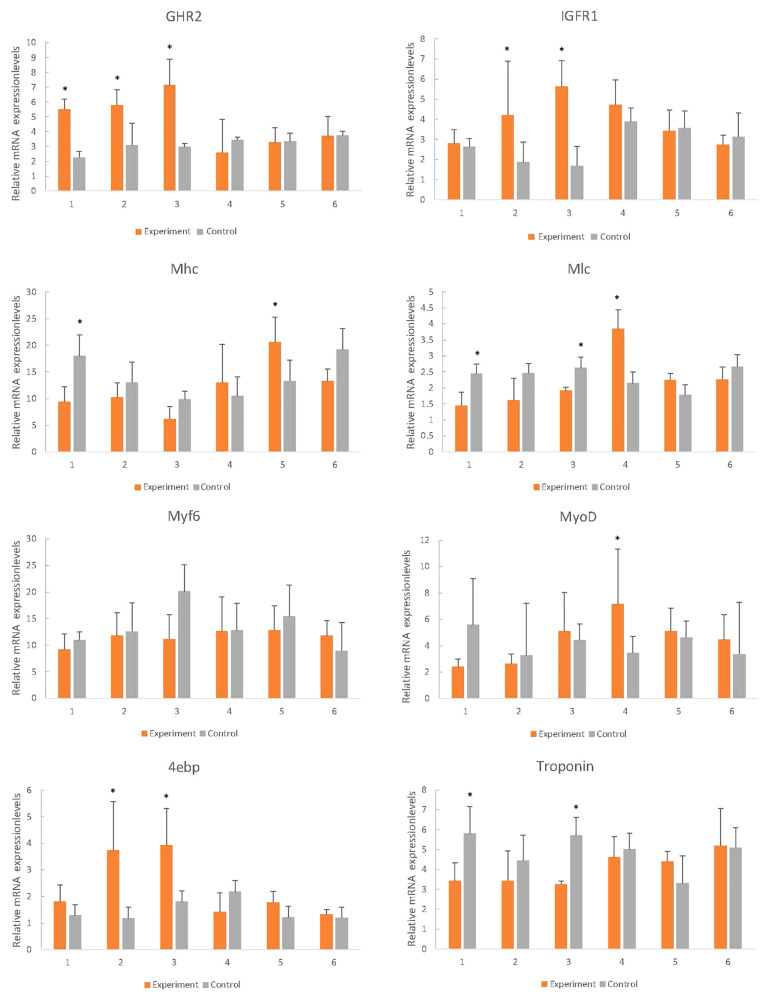
Effects of fasting and re-feeding on muscle expression of growth-related genes in mandarin fish. After acclimated for a month, fish were divided into two groups (*n* = 40/group). As for fed or re-fed group, fish were fed or fasted for 21 days, and at the end of experiment, fish were fed or re-fed for 6 h before sampling; and in the fasted group, fish had been fasted before sampling. Times of sampling: times 1, 2, 3 means 1, 2, 3 weeks of fasting respectively, times 4, 5, 6 means 1, 2, 3 weeks after re-feeding. Each bar represents the mean ± SEM (*n* = 6). Significant differences at the *P* < 0.05 level are indicated by * above the columns.

During starvation, the expression of GHR2, IGFR1 and 4ebp was up-regulated, and the difference of expression increased with the increase of starvation time, and the expression was close to that of the control group following re-feeding (Figure 7). The expression levels of Mhc, Mlc, troponin, and MyoD in starvation revealed the descent tendency, however, there was no significant difference in Myf6 expression (Figure 7).

**FIGURE 8:**
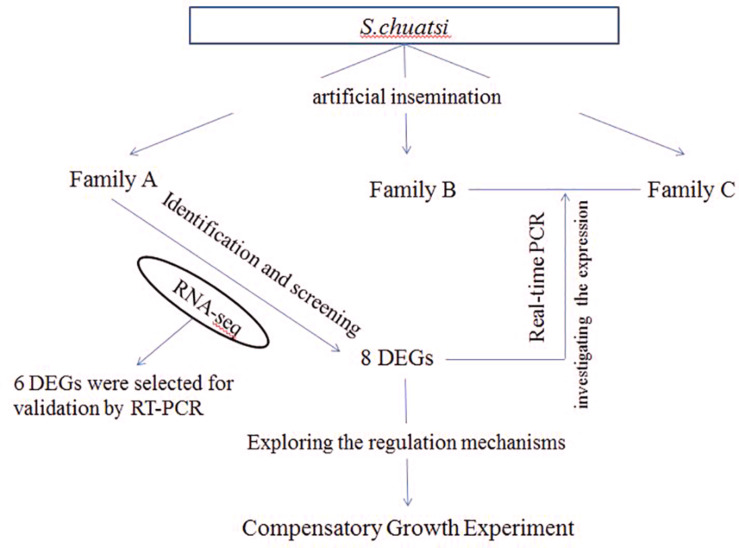
The flow chart of experimental design.

## Discussion

The growth and development of vertebrates are mainly mediated through the GH-IGF system. Especially in teleost fish, the multiple forms of GHRs, and IGFRs have been described. However, they have distinct functions and expression patterns in various fish tissues (Saera-Vila et al., 2005). In tilapia, GHR2 participates in growth and metabolism and is highly expressed in liver and muscle, while GHR1 expression is higher in liver (Pierce et al., 2007). In seabream and rainbow trout, GHR2 expression is much higher than GHR1 in numerous tissues such as muscle, pituitary, kidney, and gonad (Very et al., 2005; Jiao et al., 2006). Interestingly, although GHR expression is affected by GH in many other fish, the opposite effects are found may due to the different expression levels. The growth responses are enhanced in loaches and GH-transgenic salmon and restrained in tilapia and carp (Zhang et al., 1990; Devlin et al., 1994; Hernández et al., 1997; Nam et al., 2001). The weight and thick muscle fibers of GH/GHR-transgenic zebrafish is lower than the other genotypes (Silva et al., 2015). In our experiments, however, both the enhancement and inhibition of growth occurred under different conditions, respectively. With the change of time, the expression of GHR2 among different sizes of individuals showed no significant difference (Figure 5, GHR2), indicating that the stable expression of GHR2 was crucial to promoting growth and development. During starvation, high expression of GHR on the surface of muscle cells can save protein consumption (Pierce et al., 2007), which also has been reported in gilthead sea bream and rainbow trout (Saera-Vila et al., 2005; Gabillard and Kamangar, 2006). Therefore, in *S. chuatsi*, GHR2 appears to inhibit growth through significant high expression during starvation (Figure 7, GHR2) and plays a physiologically significant role in muscle metabolism.

IGFRs can bind to IGF-1, IGF-2 and insulin, and the affinity for insulin is much lower than for IGFs in all species (Wood and Duan, 2005). Furthermore, in contrast to the situation in mammals, the IGFR1 of fish muscle is greater abundance than insulin receptors (IR) (Wood and Duan, 2005), indicating that IGFR1 is more relevant to the regulation of muscle function than IR in fish (Reinecke et al., 2005), involving promoting growth, enhancing protein, increasing cell proliferation and reducing protein degradation (Radaelli et al., 2003). IGFR1 was highly expressed in S at 3-month old (Figure 5, IGFR1) (*P* < 0.05), suggesting IGFR1 played a more essential role for S over L in muscle, such as participating in and promoting the growth of muscle. Interestingly, prolonged fasting results in significantly reduced IGF-1 mRNA in the liver and muscle of numerous fish species (Pedroso et al., 2006; Peterson and Waldbieser, 2009; Chen et al., 2019), whereas IGFR1 increased significantly (Yakar et al., 2005; Montserrat et al., 2007; Chen et al., 2019). In this study, the IGFR1 expression markedly rose during fasting (Figure 7, IGFR1), illustrating that IGFR1 expression will increase to restrict IGF-I during starvation, which is probably related to the anti-apoptotic effects reported in mammals (Yakar et al., 2005).

Phosphorylation of protein kinase B (PKB) is controlled by the metabolic pathway regulated by IGFR1, and PKB can mediate the AKT/mTOR/p70S6K pathway to facilitate protein synthesis and cell growth (Jiang et al., 1999; Bodine et al., 2001; Sasai and Miyazu, 2010). Meanwhile, mTOR can regulate translation by 4ebp (Nissim and Nahum, 2004), and the phosphorylation of 4ebp releases eIF4E to stimulate translation initiation (Stephan et al., 2006). eIF4E is able to enhance the translation of mRNAs, implicating in cell proliferation and growth (Mamane et al., 2004), but it is hypothesized that overexpression of eIF4E leads to the deregulation of translational and cellular homeostasis (Mamane et al., 2006). However, the family of 4ebps can inhibit the assembly of eIF4F complex (Mamane et al., 2006). Thus, 4ebp inhibits cell growth and reverts the transformed phenotype of eIF4E-overexpressing cell (Mamane et al., 2006), which may save energy and prevent the malignant transformation of cells during starvation by significant expression (Figure 7, 4ebp). In conclusion, we speculate that the expression of IGFR1 will promote the expression of 4ebp through the above-mentioned pathways to cope with the food restriction. In compensatory growth experiments, the expression of IGFR1 and 4ebp was consistent (Figure 7, IGFR1 and 4ebp), which proved the hypothesis. But further research is needed on specific regulatory mechanisms. It has been reported that fasting promotes the metabolic actions of GH rather than the growth-promoting actions (Norbeck et al., 2007), however, relevant aspects of researches in GHR2, IGFR1 and 4ebp are less. In the study, the functions of GHR2, IGFR1 and 4ebp also changed from growth promotion to growth inhibition in starvation, thus highlighting the metabolic functions.

Mhc expression is significantly related with growth rate (Dhillon et al., 2010; Churova et al., 2015; Overturf and Hardy, 2015), which also affects myofiber hyperplasia and indeterminate growth (Biga and Goetz, 2006). In this study, Mhc was highly expressed in S at 5-month old (Figure 5, Mhc) (*P* < 0.05), and transcriptome results also showed that the growth rate of small fish was high, suggesting that Mhc can promote the growth of S through its high expression. In larval stage of *S. chuatsi*, the high expression of Mlc regulates muscle formation and early development (Chu et al., 2011), which also influences the tail shaft swimming character at posterior area, needing more muscle fiber and protein (Patel et al., 1996). The significantly high expression of Mlc at 5-month old in S (Figure 5, Mlc) proved that muscle protein and muscle fiber synthesize quickly to meet physiological requirement. Taken together, as important components of myosin, Mhc and Mlc played a more positive role in S comparing to L at 5-month old. Interestingly, although they all enhanced the growth of *S. chuatsi*, the function time and action modes of myosin (Mlc, Mhc), GHR2, IGRF1 and 4ebp were different. GHR2 was expressed stably to promote growth (Figure 5, GHR2), and IGFR1 had a more obvious promotion effects in S at 3-month old (Figure 5, IGFR1). Furthermore, both myosin (Mlc, Mhc) and 4ebp facilitated more markedly in S at 5-month old (Figure 5, Mlc, Mhc, and 4ebp), showing all of them had their own temporal and spatial expression patterns in *S. chuatsi*.

Myosin, the primary protein of muscles, amounts to 50% of the muscle proteins (Watabe and Ikeda, 2006). Troponin as a complex protein is largely expressed in muscle and plays a vital role in muscle contraction (Fu et al., 2009; Huang and Zhang, 2009). During fasting conditions, the markedly down-regulated of Mhc and Mlc indicated that muscle protein synthesis decreased (Figure 7, Mhc and Mlc), and muscle proteins may be used as the major energy source to maintain basic metabolism, which limited growth and was consistent with the observed reduced weight (Figure 6). In the meantime, because muscle breakdown and muscle protein synthesis were inhibited, muscle contraction and motor function were restrained, which was manifested as a significant decrease in troponin expression (Figure 7, Troponin). After re-feeding, significant up-regulation of Mhc and Mlc was observed (Figure 7, Mhc and Mlc), showing that muscle protein was no longer used as the major energy source, and synthesis of muscle and rapid compensatory growth occurred, which resulted in increased weight (He et al., 2015). As the normal synthesis and growth of muscle, muscle contraction was no longer inhibited and expression levels returned to normal (Figure 7, Troponin).

MyoD is associated with myogenesis during developmental condition which plays a regulatory role in muscle hypertrophy and muscle mass (Ahammad et al., 2015; Huang et al., 2018). Myf6 participates in myofiber differentiation by recruiting structural proteins (Xie et al., 2010). From 3- to 5-month old, MyoD and Myf6 expression had a downward trend (Figure 5, MyoD and Myf6), which agreed with the experiment phenomenon of Zhu et al. (2016). It suggested that 3-month old may be a rapid growth stage for *S. chuatsi*, accompanying with an intense myoblast differentiation, which primarily contributed to the muscle growth (Zhu et al., 2016). What puzzled us is that both MyoD and Myf6 belong to the MRFs family, but the trends of expression results were opposite in L and S (Figure 5, MyoD and Myf6), and there was no significant difference, which may be influenced by the complex regulatory systems *in vivo*. It has been reported that the reduction in muscle weight may stimulate MRFs transcription (Johansen and Overturf, 2005). And in mammals, satellite cells can express MyoD and Myf5 in response to muscle damage (Smith et al., 1994; Hawke and Garry, 2001). Interestingly, during the starvation period, the expression of MyoD fell to the lowest level and then increased following the weight loss. After re-feeding, the expression gradually decreased from the highest level, and during the whole process, the expression had a certain delayed (Figure 7, MyoD). But, the expression of Myf6 was stable throughout the process. These phenomena indicated that MyoD had sensory and regulatory effects on the weight loss and was conducive to the muscle growth and recovery for *S. chuatsi*.

## Conclusion

Numerous DEGs were identified, and several significant DEGs were chosen to explore the expression in compensatory growth. Fasting promoted the metabolic actions (growth inhibiting) of GHR2, IGFR1, and 4ebp rather than the growth-promoting actions. MyoD can sense and regulate the hunger, which also worked with Mhc and Mlc to accelerate the compensatory growth of *S. chuatsi*. This study expands our understanding of the mechanism of compensatory growth, and the elected genes will contribute to the selective breeding in future as candidate genes.

## Data Availability Statement

The original contributions presented in the study are publicly available. This data can be found in the DRYAD database: https://doi.org/10.5061/dryad.wh70rxwjx.

## Ethics Statement

The animal study was reviewed and approved by Institutional Animal Care and Ethics Committee of Sun Yat-sen University.

## Author Contributions

GL conceived the study. GL and XL designed the experiments and drafted the work or revised it critically for important content. XL, SZ, HL, and SB collected the samples. XL did the experiments. XL, SZ, and DG prepared the figures and tables. YZ, GW, XC, XZ, SL, YS, and HY made other contributions. The manuscript has been read and approved by all named authors and that there are no other persons who satisfied the criteria for authorship but are not listed. All authors contributed to the article and approved the submitted version.

## Conflict of Interest

The authors declare that the research was conducted in the absence of any commercial or financial relationships that could be construed as a potential conflict of interest.

## Abbreviations

4ebp: eukaryotic translation initiation factor 4E-binding protein
DEGs: differentially expressed genes
GH: growth hormone
GHR: GH receptors
GO: gene ontology
Hsp90 β: heat shock protein hsp90 beta
IGF: insulin-like growth factor
IGFR: insulin-like growth factor receptors
KEGG: kyoto encyclopedia of genes and genomes
L: large individuals
Mhc: myosin heavy chain
Mlc: myosin light chain
MRFs: myogenic regulatory factors
MyoD: myoblast determination protein
Myf6: myogenic factor 6
RNA-seq: RNA-sequencing
S: small individuals
SGR: specific growth rate.
